# Supplementary material for: An updated genome-scale metabolic network reconstruction of Pseudomonas aeruginosa PA14 to characterize mucin-driven shifts in bacterial metabolism
Source: NPJ Syst Biol Appl. 2021 Oct 8;7:37. doi: 10.1038/s41540-021-00198-2 (PMC8501023; doi:10.1038/s41540-021-00198-2)

iPau21 MEMOTE Report Follows

Independent Section

Contains tests that are independent of the class of modeled organism, a model's complexity or types of identifiers that are

Consistency

Stoichiometric Consistency	99.2%	X3
Mass Balance	91.2%	
Charge Balance	89.0%	
Metabolite Connectivity	100.0%	
Unbounded Flux In Default Medium	75.2%	
Sub Total	93%	X3

Annotation - Metabolites

Presence of Metabolite Annotation	100.0%
Metabolite Annotations Per Database	Info
pubchem.compound	0.0%
kegg.compound	52.7%
seed.compound	80.7%
inchikey	0.0%
inchi	0.0%
chebi	53.3%
hmdb	43.0%

Specific Section

Covers general statistics and specific aspects of a metabolic network that are not universally applicable. See readme for

SBML

SBML Level and Version	Errored
FBC enabled	Errored

Basic Information

Model Identifier	iPAU
Total Metabolites	1,310
Total Reactions	1,571
Total Genes	1,171
Total Compartments	3
Metabolic Coverage	1.34

Metabolite Information

Unique Metabolites	1,310
Duplicate Metabolites in Identical Compartments	0
Metabolites without Charge	0
Metabolites without Formula	0
Medium Components	36

bigg.metabolite	81.5%	
biocyc	51.0%	
**Metabolite Annotation Conformity Per Database**	Info	
pubchem.compound	0.0%	
kegg.compound	100.0%	
seed.compound	100.0%	
inchikey	0.0%	
inchi	0.0%	
chebi	100.0%	
hmdb	100.0%	
reactome	0.0%	
metanetx.chemical	100.0%	
bigg.metabolite	99.9%	
biocyc	100.0%	
**Uniform Metabolite Identifier Namespace**	100.0%	
**Sub Total**	75%	

Annotation - Reactions

**Presence of Reaction Annotation**	100.0%
**Reaction Annotations Per Database**	Info
rhea	29.9%

Purely Metabolic Reactions with Constraints	12
Transport Reactions	246
Transport Reactions with Constraints	1
Thermodynamic Reversibility of Purely Metabolic Reactions	0.22
Reactions With Partially Identical Annotations	0.00
Duplicate Reactions	0.00
Reactions With Identical Genes	0.47

Gene-Protein-Reaction (GPR) Associations

Reactions without GPR	52
Fraction of Transport Reactions without GPR	0.04
Enzyme Complexes	194

Biomass

Biomass Reactions Identified	1
Biomass Consistency	Errored
Biomass Production In Default Medium	3.64
Unrealistic Growth Rate In Default Medium	true
Biomass Production In Complete Medium	159.68

metanetx.reaction	45.5%	
bigg.reaction	50.4%	
reactome	0.0%	
ec-code	64.2%	
brenda	0.0%	
biocyc	30.6%	
**Reaction Annotation Conformity Per Database**	Info	
rhea	100.0%	
kegg.reaction	100.0%	
seed.reaction	89.1%	
metanetx.reaction	100.0%	
bigg.reaction	100.0%	
reactome	0.0%	
ec-code	98.9%	
brenda	0.0%	
biocyc	100.0%	
**Uniform Reaction Identifier Namespace**	100.0%	
**Sub Total**	79%	

Annotation - Genes

**Presence of Gene Annotation**	100.0%

**Ratio of Direct Metabolites in Biomass Reaction**	0.00
**Number of Missing Essential Biomass Precursors**	15

Energy Metabolism

**Non-Growth Associated Maintenance Reaction**	1
**Growth-associated Maintenance in Biomass Reaction**	true
**Number of Reversible Oxygen-Containing Reactions**	12
**Erroneous Energy-generating Cycles**	Info
MNXM3	Skipped
MNXM63	Skipped
MNXM51	Skipped
MNXM121	Skipped
MNXM423	Skipped
MNXM6	Skipped
MNXM10	Skipped
MNXM38	Skipped
MNXM208	Skipped
MNXM191	Skipped
MNXM223	Skipped
MNXM7517	Skipped
MNXM12233	Skipped

uniprot	96.8%	
ecogene	0.0%	
kegg.genes	100.0%	
ncbigi	0.0%	
ncbigene	96.8%	
ncbiprotein	96.8%	
ccds	0.0%	
hprd	0.0%	
asap	96.8%	

Gene Annotation Conformity Per Database Info

refseq	100.0%
uniprot	100.0%
ecogene	0.0%
kegg.genes	100.0%
ncbigi	0.0%
ncbigene	100.0%
ncbiprotein	100.0%
ccds	0.0%
hprd	0.0%
asap	100.0%

Sub Total 73%

MNXM89557	Skipped

Network Topology

Universally Blocked Reactions	606
Orphan Metabolites	45
Dead-end Metabolites	79
Stoichiometrically Balanced Cycles	279
Metabolite Production In Complete Medium	295
Metabolite Consumption In Complete Medium	549

Matrix Conditioning

Ratio Min/Max Non-Zero Coefficients	0.00
Independent Conservation Relations	55
Rank	1255
Degrees Of Freedom	316

Experimental Data Comparison

Growth Prediction	Skipped
Gene Essentiality Prediction	Skipped

Misc. Tests

Metabolite SBO:0000247 Presence	100.0%	
Reaction General SBO Presence	100.0%	
Metabolic Reaction SBO:0000176 Presence	99.9%	
Transport Reaction SBO:0000185 Presence	100.0%	
Exchange Reaction SBO:0000627 Presence	100.0%	
Demand Reaction SBO:0000628 Presence	Skipped	
Sink Reactions SBO:0000632 Presence	Skipped	
Gene General SBO Presence	100.0%	
Gene SBO:0000243 Presence	100.0%	
Biomass Reactions SBO:0000629 Presence	100.0%	
**Sub Total**	82%	X2
**Total Score**	85%	

Total Score

85%

Score per Category

Platform	Linux
Memote Version	0.10.2

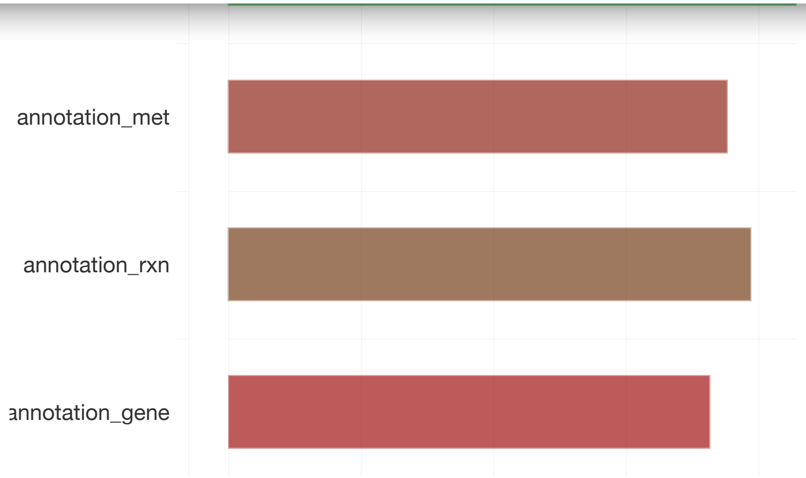
annotation_met
annotation_rxn
annotation_gene

iPau1129 MEMOTE Report Follows

Expand All   Readme   2020-07-22 14:30

Independent Section

Contains tests that are independent of the class of modeled organism, a model's complexity or types of identifiers that are used

Consistency

Stoichiometric Consistency	36.2%	x3
Mass Balance	92.4%	
Charge Balance	91.1%	
Metabolite Connectivity	100.0%	
Unbounded Flux In Default Medium	72.6%	
Sub Total	66%	x3

Annotation - Metabolites

Presence of Metabolite Annotation	0.0%
Metabolite Annotations Per Database	Info
pubchem.compound	0.0%
kegg.compound	0.0%
seed.compound	0.0%
inchikey	0.0%
inchi	0.0%
chebi	0.0%
hmdb	0.0%
reactome	0.0%

Specific Section

Covers general statistics and specific aspects of a metabolic network that are not universally applicable. See readme for more

SBML

SBML Level and Version	Errored
FBC enabled	Errored

Basic Information

Model Identifier	
Total Metabolites	1,286
Total Reactions	1,495
Total Genes	1,132
Total Compartments	2
Metabolic Coverage	1.32

Metabolite Information

Unique Metabolites	1,099
Duplicate Metabolites in Identical Compartments	0
Metabolites without Charge	3
Metabolites without Formula	5
Medium Components	33

Reaction Information

biocyc	0.0%	
**Metabolite Annotation Conformity Per Database**	Info	
pubchem.compound	0.0%	
kegg.compound	0.0%	
seed.compound	0.0%	
inchikey	0.0%	
inchi	0.0%	
chebi	0.0%	
hmdb	0.0%	
reactome	0.0%	
metanetx.chemical	0.0%	
bigg.metabolite	0.0%	
biocyc	0.0%	
**Uniform Metabolite Identifier Namespace**	100.0%	
**Sub Total**	25%	

Annotation - Reactions

**Presence of Reaction Annotation**	0.0%
**Reaction Annotations Per Database**	Info
rhea	0.0%
kegg.reaction	0.0%
seed.reaction	0.0%
metanetx.reaction	0.0%

Transport Reactions	243
Transport Reactions with Constraints	1
Thermodynamic Reversibility of Purely Metabolic Reactions	1.00
Reactions With Partially Identical Annotations	0.00
Duplicate Reactions	0.00
Reactions With Identical Genes	0.48

Gene-Protein-Reaction (GPR) Associations

Reactions without GPR	44
Fraction of Transport Reactions without GPR	0.04
Enzyme Complexes	186

Biomass

Biomass Reactions Identified	0
Biomass Consistency	Skipped
Biomass Production In Default Medium	Skipped
Unrealistic Growth Rate In Default Medium	Skipped
Biomass Production In Complete Medium	Skipped
Blocked Biomass Precursors In Default Medium	Skipped
Blocked Biomass Precursors In Complete Medium	Skipped
Ratio of Direct Metabolites in Biomass Reaction	Skipped

ec-code	0.0%	
brenda	0.0%	
biocyc	0.0%	
**Reaction Annotation Conformity Per Database**	Info	
rhea	0.0%	
kegg.reaction	0.0%	
seed.reaction	0.0%	
metanetx.reaction	0.0%	
bigg.reaction	0.0%	
reactome	0.0%	
ec-code	0.0%	
brenda	0.0%	
biocyc	0.0%	
**Uniform Reaction Identifier Namespace**	100.0%	
**Sub Total**	25%	

Annotation - Genes

**Presence of Gene Annotation**	0.0%	
**Gene Annotations Per Database**	Info	
refseq	0.0%	
uniprot	0.0%	
ecogene	0.0%	
kegg.genes	0.0%	

Energy Metabolism

**Non-Growth Associated Maintenance Reaction**	1	
**Growth-associated Maintenance in Biomass Reaction**	Skipped	
**Number of Reversible Oxygen-Containing Reactions**	12	
**Erroneous Energy-generating Cycles**	Info	
MNXM3	Skipped	
MNXM63	Skipped	
MNXM51	Skipped	
MNXM121	Skipped	
MNXM423	Skipped	
MNXM6	Skipped	
MNXM10	Skipped	
MNXM38	Skipped	
MNXM208	Skipped	
MNXM191	Skipped	
MNXM223	Skipped	
MNXM7517	Skipped	
MNXM12233	Skipped	
MNXM558	Skipped	
MNXM21	Skipped	
MNXM89557	Skipped	

ncbiprotein	0.0%	
ccds	0.0%	
hprd	0.0%	
asap	0.0%	
**Gene Annotation Conformity Per Database**	Info	
refseq	0.0%	
uniprot	0.0%	
ecogene	0.0%	
kegg.genes	0.0%	
ncbigi	0.0%	
ncbigene	0.0%	
ncbiprotein	0.0%	
ccds	0.0%	
hprd	0.0%	
asap	0.0%	
**Sub Total**	0%	

Annotation - SBO Terms

Metabolite General SBO Presence	0.0%
Metabolite SBO:0000247 Presence	0.0%
Reaction General SBO Presence	0.0%
Metabolic Reaction SBO:0000176 Presence	0.0%
Transport Reaction SBO:0000185 Presence	0.0%

Orphan Metabolites	56
Dead-end Metabolites	86
Stoichiometrically Balanced Cycles	253
Metabolite Production In Complete Medium	353
Metabolite Consumption In Complete Medium	580

Matrix Conditioning

Ratio Min/Max Non-Zero Coefficients	0.00
Independent Conservation Relations	69
Rank	1217
Degrees Of Freedom	278

Experimental Data Comparison

Growth Prediction	Skipped
Gene Essentiality Prediction	Skipped

Misc. Tests

Environment

Python Version	3.6.10
Platform	Linux
Memote Version	0.10.2

Sink Reactions SBO:0000632 Presence	Skipped	
Gene General SBO Presence	0.0%	
Gene SBO:0000243 Presence	0.0%	
Biomass Reactions SBO:0000629 Presence	Skipped	
**Sub Total**	0%	x2
**Total Score**	30%	

Total Score

30%

Score per Category

iML1515 MEMOTE Report Follows

iML1515 Expand All Readme 2020-08-03 17:51

Independent Section

Contains tests that are independent of the class of modeled organism, a model's complexity or types of identifiers that are

Consistency

Stoichiometric Consistency	100.0%	X3
Mass Balance	100.0%	
Charge Balance	100.0%	
Metabolite Connectivity	100.0%	
Unbounded Flux In Default Medium	88.9%	
Sub Total	98%	X3

Annotation - Metabolites

Presence of Metabolite Annotation	100.0%
Metabolite Annotations Per Database	Info
pubchem.compound	0.0%
kegg.compound	71.9%
seed.compound	85.3%
inchikey	0.0%
inchi	0.0%
chebi	76.7%
hmdb	56.5%

Specific Section

Covers general statistics and specific aspects of a metabolic network that are not universally applicable. See readme for

SBML

SBML Level and Version	Errored
FBC enabled	Errored

Basic Information

Model Identifier	iML1515
Total Metabolites	1,877
Total Reactions	2,712
Total Genes	1,516
Total Compartments	3
Metabolic Coverage	1.79

Metabolite Information

Unique Metabolites	1,169
Duplicate Metabolites in Identical Compartments	0
Metabolites without Charge	0
Metabolites without Formula	0
Medium Components	24

bigg.metabolite	100.0%
biocyc	79.9%
**Metabolite Annotation Conformity Per Database**	Info
pubchem.compound	0.0%
kegg.compound	100.0%
seed.compound	100.0%
inchikey	0.0%
inchi	0.0%
chebi	100.0%
hmdb	100.0%
reactome	0.0%
metanetx.chemical	100.0%
bigg.metabolite	100.0%
biocyc	100.0%
**Uniform Metabolite Identifier Namespace**	100.0%
**Sub Total**	79%

Annotation - Reactions

**Presence of Reaction Annotation**	100.0%
**Reaction Annotations Per Database**	Info
rhea	43.4%

**Purely Metabolic Reactions with Constraints**	2
**Transport Reactions**	831
**Transport Reactions with Constraints**	0
**Thermodynamic Reversibility of Purely Metabolic Reactions**	0.41
**Reactions With Partially Identical Annotations**	0.28
**Duplicate Reactions**	0.00
**Reactions With Identical Genes**	0.54

Gene-Protein-Reaction (GPR) Associations

**Reactions without GPR**	109
**Fraction of Transport Reactions without GPR**	0.06
**Enzyme Complexes**	309

Biomass

**Biomass Reactions Identified**	2
**Biomass Consistency**	Info
BIOMASS_Ec_iML1515_WT_75p37M	1.00
BIOMASS_Ec_iML1515_core_75p37M	1.00
**Biomass Production In Default Medium**	Info

metanetx.reaction	94.4%	
bigg.reaction	100.0%	
reactome	0.0%	
ec-code	40.4%	
brenda	0.0%	
biocyc	40.2%	
**Reaction Annotation Conformity Per Database**	Info	
rhea	98.1%	
kegg.reaction	100.0%	
seed.reaction	100.0%	
metanetx.reaction	100.0%	
bigg.reaction	100.0%	
reactome	0.0%	
ec-code	98.0%	
brenda	0.0%	
biocyc	100.0%	
**Uniform Reaction Identifier Namespace**	100.0%	
**Sub Total**	81%	

Annotation - Genes

**Presence of Gene Annotation**	100.0%

**Unrealistic Growth Rate In Default Medium**	Info
BIOMASS_Ec_iML1515_WT_75p37M	false
BIOMASS_Ec_iML1515_core_75p37M	false
**Biomass Production In Complete Medium**	Info
BIOMASS_Ec_iML1515_WT_75p37M	74.96
BIOMASS_Ec_iML1515_core_75p37M	74.90
**Blocked Biomass Precursors In Default Medium**	Info
BIOMASS_Ec_iML1515_WT_75p37M	1
BIOMASS_Ec_iML1515_core_75p37M	0
**Blocked Biomass Precursors In Complete Medium**	Info
BIOMASS_Ec_iML1515_WT_75p37M	0
BIOMASS_Ec_iML1515_core_75p37M	0
**Ratio of Direct Metabolites in Biomass Reaction**	Info
BIOMASS_Ec_iML1515_WT_75p37M	0.09
BIOMASS_Ec_iML1515_core_75p37M	0.13
**Number of Missing Essential Biomass Precursors**	Info
BIOMASS_Ec_iML1515_WT_75p37M	1
BIOMASS_Ec_iML1515_core_75p37M	1

Energy Metabolism

**Non-Growth Associated Maintenance Reaction**	1

uniprot	99.9%	
ecogene	99.9%	
kegg.genes	0.0%	
ncbigi	99.8%	
ncbigene	99.9%	
ncbiprotein	0.0%	
ccds	0.0%	
hprd	0.0%	
asap	99.9%	

Gene Annotation Conformity Per Database Info

refseq	0.0%
uniprot	100.0%
ecogene	100.0%
kegg.genes	0.0%
ncbigi	0.2%
ncbigene	100.0%
ncbiprotein	0.0%
ccds	0.0%
hprd	0.0%
asap	100.0%

Sub Total 63%

BIOMASS_Ec_iML1515_core_75p37M	true

Number of Reversible Oxygen-Containing Reactions 5

Erroneous Energy-generating Cycles Info

MNXM3	Skipped
MNXM63	Skipped
MNXM51	Skipped
MNXM121	Skipped
MNXM423	Skipped
MNXM6	Skipped
MNXM10	Skipped
MNXM38	Skipped
MNXM208	Skipped
MNXM191	Skipped
MNXM223	Skipped
MNXM7517	Skipped
MNXM12233	Skipped
MNXM558	Skipped
MNXM21	Skipped
MNXM89557	Skipped

Network Topology

Universally Blocked Reactions 260

Metabolite SBO:0000247 Presence	100.0%	
Reaction General SBO Presence	100.0%	
Metabolic Reaction SBO:0000176 Presence	100.0%	
Transport Reaction SBO:0000185 Presence	98.8%	
Exchange Reaction SBO:0000627 Presence	100.0%	
Demand Reaction SBO:0000628 Presence	100.0%	
Sink Reactions SBO:0000632 Presence	Skipped	
Gene General SBO Presence	100.0%	
Gene SBO:0000243 Presence	100.0%	
Biomass Reactions SBO:0000629 Presence	100.0%	
Sub Total	91%	x2
Total Score	91%	

Total Score

91%

Score per Category

Stoichiometrically Balanced Cycles	61
Metabolite Production In Complete Medium	181
Metabolite Consumption In Complete Medium	233

Matrix Conditioning

Ratio Min/Max Non-Zero Coefficients	0.00
Independent Conservation Relations	31
Rank	1845
Degrees Of Freedom	867

Experimental Data Comparison

Growth Prediction	Skipped
Gene Essentiality Prediction	Skipped

Misc. Tests

Environment

Python Version	3.6.10
Platform	Linux
Memote Version	0.10.2

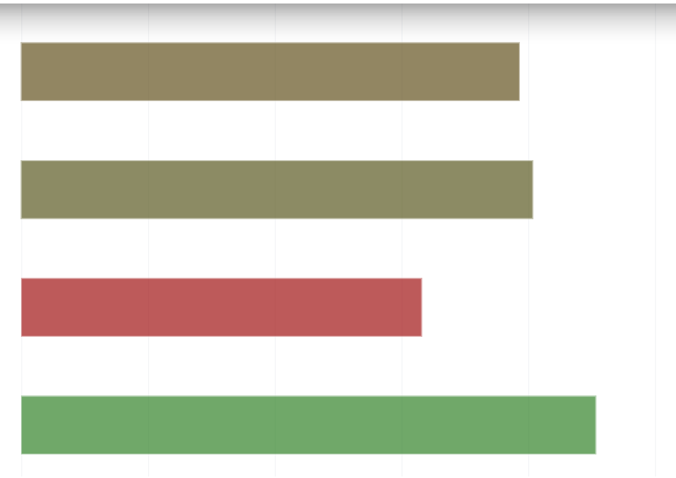

Supplement: Supplementary file 7 — Supplementary Information [file 41540_2021_198_MOESM7_ESM.pdf]
